# Neuromuscular Attributes are Associated with Poor Mobility in Older Adults with Vascular Risk Conditions

**DOI:** 10.14283/jfa.2019.42

**Published:** 2019-11-26

**Authors:** Azizah J. Jor'dan, M.E. Jacob, E. Leritz, J.F. Bean

**Affiliations:** 1 New England Geriatric Research, Education, and Clinical Center, VA Boston Healthcare System, Boston, MA, USA; 2 Department of Psychiatry, Harvard Medical School, Boston, MA, USA; 3 Department of Physical Medicine & Rehabilitation, Harvard Medical School, Boston, MA, USA; 4 School of Public Health, Boston University, Boston, MA, USA; 5 Spaulding Rehabilitation Hospital, Boston, MA, USA; 6 New England GRECC, 150 South Huntington Ave, 02130, Boston, MA, USA

**Keywords:** Vascular risk, mobility, neuromuscular attributes, cardiovascular disease

## Abstract

**Background:**

The mobility of older adults is limited by the compounding effects of vascular health conditions, or vascular risk burden. However, little is known about the role of neuromuscular attributes among those in which vascular risk burden contributes to mobility limitations.

**Objective:**

We investigated (1) the relationship between the absence/presence of type 2 diabetes, hypertension, and/or obesity and mobility measures and neuromuscular attributes, and (2) whether the association between vascular risk burden and mobility is mediated by lower limb neuromuscular attributes.

**Design:**

Cross-sectional analysis of baseline data from 430 older adults within the Boston RISE Study.

**Measurements:**

Measures of mobility were the Short Physical Performance Battery, habitual gait speed, and functional mobility as measured by the Late Life Function Instrument. We also evaluated lower limb neuromuscular attributes, namely leg strength, leg velocity, trunk extensor muscle endurance, knee and ankle range of motion, and sensory loss.

**Results:**

Participants self-reported the presence of None (n=93), One (n=179), Two (n=114), or Three (n=44) of the following conditions: diabetes, hypertension, and obesity. Multivariable regression models indicated that those with a greater vascular risk burden had worse performance on the Short Physical Performance Battery (p=0.01), slower gait speed (p=0.0003) and lower Basic and Advanced Late Life Function Instrument scores (p<0.003). These associations were independent of multiple covariates. Vascular risk burden was also found to be negatively associated with leg strength (p=0.0002) and knee flexion range of motion (p<0.0001) and an associated non-significant trend was observed with leg velocity (p=0.06). In addition, the association between vascular risk burden and mobility outcomes were found to be partially mediated by leg strength, leg velocity, and knee flexion range of motion.

**Conclusions:**

Among older adults with vascular risk burden and mobility problems, neuromuscular impairments in attributes such as leg strength, leg velocity, and knee range of motion may need to be treatment priorities.

## Introduction

Mobility is important in the lives of older adults as it helps to maintain independence. The decline in mobility has been separately linked to vascular risk factors such as obesity, hyperglycemia, hyperlipidemia, and hypertension in older adults (1, 2). For example, diabetes and hypertension are independently linked to slow gait speed, a metric of mobility and a vital indicator of mortality (1). An increasing number of vascular risk components (i.e., hypertension, high C-reactive protein, obesity, diabetes, and smoking) has been shown to linearly increase the likelihood of gait speed decline, and disability among older adults (2). In parallel, other studies have independently linked poor mobility outcomes to lower limb neuromuscular attributes, such as leg strength and velocity, trunk muscle endurance, and range of motion (ROM) among older adults (3). There is also evidence suggesting that Metabolic Syndrome (i.e., the presence of three or more of the following: hypertension, hyperglycemia, hyperlipidemia, and abdominal obesity), is associated with lower limb weakness among men (4). However, it is not known if the compounding of vascular risk, or vascular risk burden (VRB), affects other lower limb neuromuscular attributes.

Vascular risk factors rarely occur in isolation (5), and it is not evident how the co-existence of multiple vascular conditions, or the VRB, influences mobility in older adults. Additionally, the mechanisms by which VRB influence mobility are not clear.

Therefore, the aim of this study was to determine the link between VRB and 1) mobility as assessed by the Short Physical Performance Battery (SPPB), habitual gait speed, and the Late Life Function Instrument (LLFI); and 2) lower limb neuromuscular attributes (e.g., leg strength and leg velocity) and to determine whether the potential associations between VRB and mobility measures were mediated by lower limb neuromuscular attributes. We hypothesized that older adults with a greater VRB will have poorer mobility performance and be deficient in lower limb neuromuscular attributes. We further hypothesized that lower limb neuromuscular factors that are associated with VRB status, will mediate the association between VRB and mobility.

## Methods

### Study Design

This cross-sectional study used the data from the 430 community-dwelling primary care patients who participated in the Boston Rehabilitative Impairment Study of the Elderly (Boston RISE) and were examined at baseline between December 2009 and January 2012. This prospective cohort study aimed to identify modifiable neuromuscular impairments that are associated with mobility decline in older adults. The design and methodology for this study has been previously published in detail (6). Briefly, to be included, individuals had to be ≥65 years of age, able to understand and communicate in English, and have difficulty or task modification with walking half a mile or climbing one flight of stairs. The exclusion criteria were: 1) presence of a terminal disease (e.g. receiving hospice services, metastatic cancer), 2) major surgery or myocardial infarction in the last 6 months, 3) planned major surgery, 4) planned move from the area within 2 years, 5) Mini-Mental Status Exam (MMSE) score <18, or 6) major medical problems that interfered with safe and successful testing (e.g., history hip replacement with recurrent dislocation, uncontrolled hypertension, loss of lower extremity). All participants provided written informed consent as approved by the Institutional Review Board of Spaulding Rehabilitation Hospital.

### Data Collection

Socio-demographic characteristics (age, sex, race, education) and disease status (type 2 diabetes and hypertension) were self-reported using a comorbidity questionnaire that consisted of 17 frequent general practice conditions for which patients were receiving treatment and/or had limitations on their activities (7). Study staff measured height and weight using standardized techniques, calculated BMI, and classified participants as obese if their BMI was 30 or higher. Depression severity was assessed using the Patient Health Questionnaire-9 (PHQ-9), and cognitive ability was assessed using the MMSE.

#### Mobility Measures

Mobility measures consisted of the following: 1) SPPB, a reliable and valid screening test used to characterize lower extremity function (8). The test includes measures of progressive standing balance, habitual walking speed, and chair stand ability; 2) gait speed, a sensitive measure for assessing functional status and overall health. This measure was collected during two 4-meter usual pace walking trials from the SPPB; and 3) the Basic and Advanced Lower Limb Function subdomains of the LLFI, a validated questionnaire that captures the participant's perceived ability to do specific aspects of their daily routines (function) and is a component of the larger Late Life Function and Disability Instrument (9). Given the aim of the current study, which focused on mobility outcomes, we evaluated scores on the Basic and Advanced lower extremity function subdomains of the LLFI.

#### Neuromuscular Measures

Leg strength was measured by determining the 1 repetition maximum (1RM) for each leg with a Keiser pneumatic leg press machine using a previously published protocol (10). Peak leg strength was defined as the maximum value observed on either side. Peak power was defined as the highest recorded power out of five trials performed with each leg at 40% and 70% of the 1RM. Peak leg velocity was calculated by dividing peak power by the graphically displayed force at peak power recorded during the testing. Trunk extensor muscle endurance (11) was measured as the length of time in seconds (up to 150 seconds) that the participant was able to maintain their trunk in a neutral position within the sagittal plane in line with their pelvis and legs with arms crossed against the chest. Knee and ankle ROM were measured with a goniometer (12). Ankle ROM was considered to be impaired if there was inability to dorsiflex past 90° or plantar flex past 110° in either leg. Foot sensation was measured over the dorsum of the big toes using the Semmes-Weinstein monofilament test. Both impaired ankle ROM and sensory loss were dichotomized as being present or absent.

### Statistical Analysis

Participants were classified into VRB groups (i.e., None, One, Two or Three condition[s]) based on the self-reported absence or presence of type 2 diabetes, hypertension, and obesity. VRB groups were characterized using means and standard deviations for continuous variables and frequency (percentage) for categorical data. The Kruskal-Wallis test was used to determine if the group demographics, PHQ-9, MMSE, mobility measures, and the neuromuscular attributes differed by VRB.

Multivariable regression analyses were used to examine the association between VRB (predictor) and 1) mobility measures (i.e., SPPB total score, gait speed, Basic and Advanced LLFI), and 2) neuromuscular attributes (leg strength, leg velocity, trunk extensor muscle endurance, knee flexion ROM, ankle ROM, and sensory loss). Separate models were developed for each outcome and were adjusted for the pre-specified confounders of age, race, sex, PHQ-9, and MMSE scores. Mediation analysis using regression was subsequently conducted to determine whether the potential associations between VRB and mobility measures were in part mediated by neuromuscular attributes. Attributes that demonstrated 1) a significant association with VRB or 2) a clinically meaningful group difference, as determined by previous literature, were introduced into mobility measure regression models. A mediator effect was defined as a reduction in the estimate by >10% when the neuromuscular attribute was included in the regression model (13, 14). Significance levels were set to p<0.05. All analyses were performed using JMP software (SAS Institute, Cary, NC).

## Results

Group demographics, mobility, and neuromuscular characteristics are listed in Table 1. Those with no vascular conditions represented 22% of the cohort, 42% had one vascular condition, 26% had two vascular conditions, and 10% had three vascular conditions. The groups were similar in sex, race, education, and MMSE scores. Gait speed, Basic and Advanced LLFI, lower leg strength, and knee flexion ROM differed by VRB group.

**Table 1:** Complete population demographic and clinical characteristics

		VRB			
	None N=93	One N=179	Two N=114	Three N=44	P
Age, years	77.4 (6.8)	78.0 (7.2)	75.3 (6.7)	72.3 (4.9)	<0.001*
Female, %	65.6	68.2	65.8	75.0	0.69
Non-white, %	11.8	17.3	17.5	29.6	0.09
BMI, kg/m2	25.6 (2.8)	27.3 (4.5)	33.6 (6.4)	36.2 (5.4)	<0.001*
Education, %<HS	14.0	11.2	13.2	13.6	0.14
PHQ-9	1.5 (3.9)	1.3 (3.0)	1.1 (2.4)	1.6 (3.4)	0.88
Mini-mental State Exam	27.5 (2.3)	27.5 (2.2)	27.2 (2.5)	27.4 (2.7)	0.68
Mobility Measures					
SPPB Total	8.8 (2.4)	8.7 (2.3)	8.8 (2.1)	8.0 (2.1)	0.14
Gait Speed, m/s	0.94 (0.2)	0.90 (0.2)	0.91 (0.2)	0.83 (0.2)	0.02*
Basic LLFI	66.9 (12.7)	66.6 (12.2)	67.0 (11.9)	59.5 (9.6)	<0.001*
Advanced LLFI	44.3 (13.0)	43.8 (14.9)	40.1 (12.5)	32.7 (18.5)	<0.001*
Neuromuscular Attributes					
Leg Strength, kg	9.7 (2.3)	9.7 (2.7)	9.3 (2.4)	8.2 (2.2)	0.002*
Leg Velocity, m/s	0.99 (0.2)	1.01 (0.2)	1.01 (0.3)	0.93 (0.3)	0.34
Trunk Extension Endurance, s	100.1 (55.5)	95.6 (58.7)	93.9 (60.5)	88.2 (61.1)	0.73
Knee Flexion, deg	128.6 (9.8)	127.4 (12.5)	120.0 (14.8)	116.8 (15.5)	<0.001*
Ankle ROM Impairment, % yes	23.7	32.0	23.7	36.4	0.19
Sensory Loss, % yes	33.7	26.3	27.7	34.9	0.49


Data = Mean (SD); p < 0.05 Kruskal-Wallis; None = no vascular risk symptoms; One = one vascular risk symptom; Two = 2 vascular risk symptoms/obesity; Three = 3 vascular risk symptoms/obesity; PHQ — Patient Health Questionnaire; SPPB — Short Physical Performance Battery; LLFI — Late Life Function Instrument; ROM — range of motion; Normal mean/range: SPPB (for average age 75 years) — women, 7.79 ± 3.22 and men, 9.03 ± 3.12; Gait speed (for range age 70–79 years) — women, 1.13 m/s and men, 1.26 m/s; MMSE — score of ≥24 denotes normal cognition; PHQ-9 Depression — score of 0–4 mean «none to minimal» depression.

Regression analysis determined that VRB status was linked to performance on the SPPB (p=0.01), gait speed (p=0.0003), and lower Basic (p=0.003) and Advanced LLFI scores (p<0.0001) (Table 1), such that those with a greater VRB had worse performance on these mobility measures, after adjusting for age, race, sex, PHQ-9, and MMSE scores (Table 3, Model 1).

**Table 3:** Separate Multivariable Regression Models Examining Mediation of the Association between Vascular Risk Burden (VRB) and Mobility by Leg Strength, Knee Flexion ROM, or Leg Velocity

MOBILITY MEASURES	VRB GROUP	MODEL 1 ESTIMATE (95% CI)	P	MODEL 2 ESTIMATE (95% CI)	P	MODEL 3 ESTIMATE (95% CI)	P	MODEL 4 ESTIMATE (95% CI)	P
SPPB Total			0.01*		0.49		0.10		0.05
	None	1 [Reference]		1 [Reference]		1 [Reference]		1 [Reference]	
	One	0.32 (−0.007, 0.641)	0.05	0.05(−0.257, 0.362)	0.74†	0.20(−0.139, −0.013)	0.23†	0.22(−0.101, 0.545)	0.18†
	Two	0.20 (−0.164, 0.554)	0.29	0.16(−0.183, 0.498)	0.36†	0.26(−0.080, 0.632)	0.13	0.12(−0.232, 0.478)	0.50†
	Three	−0.85(−1.367, −0.342)	0.001	−0.38(−0.876, 0.120)	0.14†	−0.63 (−1.148, −0.116)	0.02†	−0.71(−1.223, −0.196)	0.01†
Gait Speed			0.0003*		0.04*		0.03*		0.004*
	None	1 [Reference]		1 [Reference]		1 [Reference]		1 [Reference]	
	One	0.03 (−0.003, 0.055)	0.08	0.008(−0.022, 0.037)	0.61†	0.009(−0.020, 0,038)	0.54†	0.01 (−0.018, 0.039)	0.47†
	Two	0.005 (−0.027, 0.038)	0.74	0.005(−0.028, 0.037)	0.76	0.017(−0.014, 0.048)	0.29	0.004(−0.028, 0.036)	0.80†
	Three	−0.09 (−0.138, −0.046)	<0.001	−0.06(−0.107, −0.011)	0.02†	−0.065(−0.111, −0.019)	0.005†	−0.07(−0.118, −0.026)	0.002†
Basic LLFI			0.003*		0.07		0.02*		0.005*
	None	1 [Reference]		1 [Reference]		1 [Reference]		1 [Reference]	
	One	1.74 (−0.035, 3.511)	0.05	0.77(−1.013, 2.552)	0.40†	1.05(−0.738, 2.844)	0.25†	1.62(−0.248, 3.487)	0.09
	Two	1.77 (−0.193, 3.734)	0.07	2.07 (0.110, 4.030)	0.04	2.29 (0.338, 4.233)	0.02	2.16 (0.100, 4.210)	0.04
	Three	−5.36 (−8.167, −2.562)	0.0002	−3.57 (−6.436, −0.695)	0.02†	−4.10(−6.922, −1.273)	0.005†	−5.37(−8.338, −2.399)	0.0004
Adv LLFI			<0.0001*		0.004*		0.003*		<0.0001*
	None	1 [Reference]		1 [Reference]		1 [Reference]		1 [Reference]	
	One	4.13 (2.046, 6.217)	0.0001	2.35 (0.396, 4.309)	0.02†	2.71 (0.666, 4.762)	0.01†	3.57 (1.423, 5.723)	0.001†
	Two	−0.39 (−2.701, 1.918)	0.74	−0.52(−2.668, 1.635)	0.64	0.56(−1.670, 2.785)	0.62†	−0.61 (−2.973, 1.758)	0.614
	Three	−8.24 (−11.539, −4.946)	<0.0001	−4.85(−8.00, −1.702)	0.003†	−5.85(−9.083, −2.624)	0.0004†	−7.48(−10.896, −4.057)	<0.0001


Model 1 adjusted for age, race, sex, PHQ-9, and MMSE Scores; Model 2 = Model 1 + leg strength; Model 3 = Model 1 + knee flexion range of motion (ROM); Model 4 = Model 1 + leg velocity; (†) = >10 % change in estimate from Model representing significant mediation.

As shown in Table 2, those with a greater VRB were shown to have higher impairment in lower leg strength (p=0.0002) and knee flexion ROM (p<0.0001), independent of age, race, sex, PHQ-9, and MMSE scores. Of note, although statistically significant, the variation specifically explained by the knee flexion model is weak. Leg velocity did not achieve statistical significance (p=0.06), however, the observed differences between groups (0.09m/s) exceeded what is defined as a clinically meaningful threshold in the literature (15) and leg velocity was therefore included in subsequent mediation analyses. Trunk extension muscle endurance, ankle ROM, or sensory loss did not differ by VRB status (Table 2).

**Table 2:** Separate Univariate and Multivariable Regression Models Evaluating the Association between Vascular Risk Burden (VRB) and Measures of Mobility and Neuromuscular Attributes

	Model	Statistics^a^	Model	Statistics^b^			VRB		
	R^2^	P	R^2^	P	None N=93	One N=179	Two N=114	Three N=44	P
OUTCOMES									
*Mobility Measures*									
SPPB Total	0.01	0.218	0.14	<0.0001	8.9 ± 0.2	8.8 ± 0.2	8.7 ± 0.2	7.7 ± 0.3	0.01*
Gait Speed, m/s	0.02	0.038	0.23	<0.0001	0.96 ± 0.2	0.92 ± 0.1	0.90 ± 0.02	0.80 ± 0.03	0.0003*
Basic LLFI	0.03	0.003	0.11	<0.0001	67.5 ± 1.2	67.4 ± 0.9	67.4 ± 1.1	60.3 ± 1.8	0.003*
Advanced LLFI	0.06	<0.0001	0.16	<0.0001	45.8 ± 1.4	45.5 ± 1.1	40.9 ± 1.3	33.1 ± 2.1	<0.0001*
*Neuromuscular Attributes*									
Leg Strength, kg	0.03	0.005	0.21	<0.0001	10.1 ± 0.2	10.2 ± 0.2	9.5 ± 0.2	8.4 ± 0.4	0.0002*
Leg Velocity, m/s	0.009	0.358	0.29	<0.0001	1.04 ± 0.02	1.06 ± 0.02	1.04 ± 0.02	0.95 ± 0.04†	0.06
Trunk Extension Endurance, s	0.003	0.742	0.06	0.0018	102.6 ± 6.2	98.8 ± 4.6	94.2 ± 5.7	85.2 ± 9.4	0.43
Knee Flexion, deg	0.10	<0.0001	0.12	<0.0001	128.9 ± 1.4	127.9 ± 1.0	120.0 ± 1.2	116.2 ± 2.0	<0.0001*
Ankle ROM Impairment, % yes^c^	0.01	0.189	0.03	0.0847	24.6 ± 0.05	32.8 ± 0.04	25.7 ± 0.04	40.8 ± 0.070	0.14
Sensory Loss, % yes^c^	0.006	0.493	0.10	<0.0001	34.6 ± 0.05	28.2 ± 0.03	32.2 ± 0.04	46.3 ± 0.07	0.12


Model Statistics^a^ — Univariate R^2^ and p-value for the overall regression model; Model Statistics^b^ — Multivariable R^2^ and p-value for the overall regression model; VRB − Data = Least Square Means ± SE; (*) p < 0.05 for VRB indicator variable in the multivariable regression model; (†) clinically meaningful difference; None = no vascular risk symptoms; One = one vascular risk symptom; Two = 2 vascular risk symptoms/obesity; Three = 3 vascular risk symptoms/obesity; SPPB — Short Physical Performance Battery; LLFI — Late Life Function Instrument; ROM — range of motion; All models adjusted for age, race, sex, PHQ-9, and Mini-mental Exam Scores; ^c^ Logistic Regression

Regression analysis was used to test whether VRB status predicted mobility (i.e., SPPB, gait speed, Basic and Advanced LLFI) when significant and clinically meaningful lower limb neuromuscular attributes (i.e., leg strength, knee flexion, and leg velocity) were included as potential mediators. As shown in Table 3, when lower leg strength was included in the subsequent models (Model 2), VRB was no longer associated with SPPB (p=0.49) or Basic LLFI (p=0.07). When leg strength was added to the model, VRB coefficients were attenuated by 20–84% for SPPB, 33–73% for gait speed, 33–56% for Basic LLFI, and 41–43% for Advanced LLFI models.

When knee flexion ROM was added to the model (Model 3), VRB was no longer associated with SPPB (p=0.10). Additionally, VRB coefficients were attenuated by 26–38% for SPPB, 28–70% for gait speed, 24–40% for Basic LLFI, and 29–243% for Advanced LLFI models.

Model 4 shows results for the inclusion of leg velocity in the model. VRB was no longer associated with SPPB (p=0.05) and VRB coefficients were attenuated by 17–40% for SPPB and 20–67% for gait speed. Additionally, the coefficient for VRB category ONE was attenuated by 14% when leg velocity was added to the Advanced LLFI model (Table 3).

## Discussion

This analysis found that VRB status was negatively associated with mobility, such that greater vascular burden is linked to greater limitation on both performance-based and patient-reported measures of mobility. Additionally, VRB status was associated with higher impairment in leg strength, leg velocity, and knee ROM. We also found that these same neuromuscular attributes partially mediated the association between VRB status and mobility measures, independent of age, race, sex, PHQ-9, and MMSE scores.

Multi-morbidity of metabolic conditions puts individuals at a higher risk for developing mobility limitations (2). Our study demonstrated that VRB, as defined by the absence or presence of type 2 diabetes, hypertension, and obesity, was associated with worse performance on the SPPB, slower gait speed, and lower scores on the Basic and Advanced LLFI. Our findings are consistent with other research addressing mobility and metabolic syndrome as well as vascular risk factors for coronary heart disease and cardiovascular disease. For example, a study of metabolic syndrome by Penninx et al observed that older adults with metabolic syndrome had a 50% higher chance of developing future mobility limitations (16).

Another important finding from this study is that VRB status was linked to certain neuromuscular attributes. Individuals with the greatest VRB (i.e., three) had lower leg strength, compared to those with less burden. Additionally, there was a weak, yet significant association between VRB and knee flexion, such that those with two or three vascular risks had a lower degree of knee flexion ROM, compared to those with no or one vascular risk. Post hoc comparison of VRB status and leg velocity determined that those with three vascular risks manifested significantly slower leg velocity, compared to those with one vascular risk. Trunk muscle endurance may also have clinical relevance (11), but this association was difficult to detect given the small size of the Three VRB group and diversity of the vascular risks within each group. However, other studies have shown independent links between type 2 diabetes, obesity, or hypertension and neuromuscular consequences (17, 18, 19). For example, studies report that regardless of neuropathy, diabetic patients as well as those who are obese have decreased lower extremity muscle strength and power measures (20, 21, 22).

One longitudinal study found a 50% accelerated skeletal muscle decline in those with type 2 diabetes, compared to those without, while another study reported that obesity was linked to a four-fold higher incidence of developing knee osteoarthritis, which impairs knee ROM (20, 23). These findings are supported by our results which suggest that lower leg strength, leg velocity, and knee flexion ROM is linked to the burden of underlying vascular disturbances.

The capacity to perform independent functioning depends upon the integrity of the varied body systems that underlie performance. In the current study, leg strength, leg velocity, and knee ROM were all linked to mobility skills among primary care patients with varying VRB status. The exact linkage between vascular/metabolic conditions and the manifestation of mobility limitations is not known. However, this association may be due to the vascular damage both centrally in the brain and in the periphery caused by type 2 diabetes, hypertension, and obesity. These vascular changes can manifest as reduced arterial wall compliance, arterial thickness and stiffening, endothelial dysfunction, and impaired relaxing and contracting mechanisms which leads to peripheral vascular resistance (24, 25, 26). These impairments also support studies associating vascular risk conditions with brain atrophy and reduced blood flow in the brain (27, 28). Moreover, our findings build upon prior research focusing upon mobility problems in general and among individuals with peripheral artery disease (3, 19, 29). When taken together, vascular risk conditions cause inadequate supply of blood and oxygen levels to meet the metabolic demands associated with exercises as well as other physical activities.

Higher vascular risk burden is often associated with aging, lower cognitive functioning, higher BMI and mortality rates (30, 31, 32). Interestingly, MMSE scores were not able to differentiate VRB within this cohort. This could be due to the variation in group sample size, group differences in age, and/or the protective factor of education. However, BMI was indeed able to differentiate the VRB groups, such that those with the highest VRB were those with the highest BMI. Within the current study, the group with the highest VRB was significantly younger than those with no VRB. This is could be due to the smaller sample of Three VRB compared to the None VRB groups. Additionally, the None group consisted of a wider age range (66–91) compared to the Three VRB group (65–83). We speculate that those with no VRB are living longer and ultimately are able to be studied, while those with higher VRBs, whom are at higher risk for mortality, are not.

Our study has several limitations. Due to its cross-sectional design, we could not determine causality. This was not a population-based sample, and data collection was confined to a specific single healthcare system within the study area, thus our findings may not be generalizable to a more racially or ethnically diverse population in a different region of the country. The vascular conditions utilized were self-reported and obesity was clinically assessed, as opposed to the more specific metabolic syndrome condition of abdominal obesity. Additionally, data on hyperlipidemia was not collected in the parent study (Boston RISE). Lastly, neuropathy, specific to type 2 diabetes, was not included in the data collection; however, sensory loss was assessed as a variable of interest for all participants. The strengths of this study include extensive data collection of neuromuscular impairment status and severity in a large sample of older adult primary care patients with mobility difficulty. Outcomes measures in the current reflect a cohort that may be frail (e.g., low SPPB scores). Although we did not specifically measure frailty, our findings are likely to apply to frail older adults as well. Furthermore, all study measures were well established, valid, and reliable among older adults (6) and all of the neuromuscular attributes we studied are clinically relevant and prioritized within rehabilitative care.

In conclusion, our results suggest that greater VRB is associated with impaired neuromuscular integrity, specifically leg strength and velocity and knee ROM, which is associated with the manifestation of mobility limitation in older adults. These findings underscore the relevance of peripheral neuromuscular attributes that underlie mobility limitations among patients with VRB and, therefore, should be considered in rehabilitative treatment.

*Funding:* This work is supported by the National Institutes of Health (R01 grant number AG032052-03 and K24 grant number HD070966-01) and the National Center for Research Resources (grant number UL1RR025758-01). Manuscript preparation was supported by the National Institutes of Health (K99 grant number AG051766) awarded to A.J.J.

*Conflict of interest:* The authors report no disclosures.

*Ethical standards:* The procedures followed were in accordance with the ethical standards of the Institutional Review Board and with the Helsinki Declaration of 1975.
